# Controlling osteoblast morphology and proliferation via surface micro-topographies of implant biomaterials

**DOI:** 10.1038/s41598-020-69685-6

**Published:** 2020-07-30

**Authors:** Kerstin Rabel, Ralf-Joachim Kohal, Thorsten Steinberg, Pascal Tomakidi, Bernd Rolauffs, Erik Adolfsson, Paola Palmero, Tobias Fürderer, Brigitte Altmann

**Affiliations:** 1 Department of Prosthetic Dentistry, Center for Dental Medicine, Medical Center - University of Freiburg, Faculty of Medicine, University of Freiburg, Hugstetterstr. 55, 79106 Freiburg, Germany; 2 Department of Oral Biotechnology, Center for Dental Medicine, Medical Center - University of Freiburg, Faculty of Medicine, University of Freiburg, Hugstetterstr. 55, 79106 Freiburg, Germany; 3 G.E.R.N Center for Tissue Replacement, Regeneration & Neogenesis, Department of Orthopedics and Trauma Surgery, Medical Center - University of Freiburg, Faculty of Medicine, University of Freiburg, Engesserstr. 4, 79108 Freiburg, Germany; 4 Division Materials and Production - RISE IVF AB, RISE Research Institutes of Sweden, Argongatan 30, 43153 Mölndal, Sweden; 5 Department of Applied Science and Technology, INSTM R.U. PoliTO, LINCE Lab., Politecnico Di Torino, Corso Duca Degli Abruzzi, 24, 10129 Turin, Italy; 6 MOESCHTER GROUP Holding GmbH & Co. KG, Hesslingsweg 65 - 67, 44309 Dortmund, Germany; 7 G.E.R.N Center for Tissue Replacement, Regeneration & Neogenesis, Department of Prosthetic Dentistry, Medical Center - University of Freiburg, Faculty of Medicine, University of Freiburg, Engesserstr. 4, 79108 Freiburg, Germany

**Keywords:** Cell biology, Preclinical research, Biomarkers, Implants

## Abstract

Current research on surface modifications has yielded advanced implant biomaterials. Various implant surface modifications have been shown to be promising in improving bone target cell response, but more comprehensive studies whether certain implant surface modifications can directly target cell behavioural features such as morphogenesis and proliferation are needed. Here, we studied the response of primary alveolar bone cells on various implant surface modifications in terms of osteoblast morphology and proliferation in vitro. Analyses of surface modifications led to surface-related test parameters including the topographical parameters micro-roughness, texture aspect and surface enlargement as well as the physicochemical parameter surface wettability. We compared osteoblast morphology and proliferation towards the above-mentioned parameters and found that texture aspect and surface enlargement but not surface roughness or wettability exhibited significant impact on osteoblast morphology and proliferation. Detailed analysis revealed osteoblast proliferation as a function of cell morphology, substantiated by an osteoblast size- and morphology-dependent increase in mitotic activity. These findings show that implant surface topography controls cell behavioural morphology and subsequently cell proliferation, thereby opening the road for cell instructive biomaterials.

## Introduction

To improve the interactions between the biomaterial and the cells of the target tissue the fabrication of defined surface properties is of importance. In oral implantology the impact of biomaterial surface properties such as topography and chemistry on bone tissue response has already been recognized in the last decades of the twentieth century^1^. In regard to these properties, numerous surface modifications of implant materials have been developed to ensure a long-lasting bone-to-implant interface (reviewed in^2,3^). Modifications of the above-mentioned properties, i.e. surface topography and chemistry are common, since it has been shown that they influence cell behaviour in vitro and implant integration into bone in vivo (for review see^4^). With respect to surface topography, it is currently accepted that microrough implant surfaces are superior over smooth surfaces to support osteoblast-triggered hard tissue integration^5^. In this context, various surface modification methods have been developed to roughen the metallic or ceramic implant surfaces. Although the different modification techniques are suitable to create the necessary average surface roughness, they yield different micro- and nanotopographical surface characteristics, which lead to different cellular reactions^6^. Together with the results from clinical studies which report a lack of beneficial effect of implant surface roughness on the long-term preservation of the peri-implant marginal bone level^7^, these findings suggest that other details of the surface structure can be more determining on cell-biomaterial interaction than their roughness degree^8^. In this context, the work of several studies suggests that distinct structural micro- and/or nanoscale features, which arise from the different surface modification techniques, may play a significant role in influencing target cell behaviour and thus ultimately define the quality of bone integration^9–20^. Despite plenty preclinical in vitro and in vivo studies on the evaluation of new implant biomaterials, there still exists sparse knowledge on the link between specific surface characteristics and the cell response at the tissue-implant interface^21^. However, such knowledge would greatly enhance our capability to rationally design novel biomaterials for instructing cell behaviour^17^.

The osteoblast response to an implant surface is generally characterized by an initial cell attachment and spreading phase, followed by the proliferation and subsequent differentiation of the cells, which finally leads to direct bone apposition to the biomaterial surface^4^. Regarding this issue, an important aspect is that according to Anselme et al.^4,22^ the quality of the first phase of cell-biomaterial interaction affects the quality of the second phase, meaning that stable cell adhesion is essential for further proliferation and differentiation. The classical understanding of this interconnection of both phases is based on the notion, that adhesion-dependent cells will only produce growth and differentiation factors if adhesion to an extracellular or engineered matrix can be achieved^23^. An often neglected point in this context is that not only secreted factors influence further cell behaviour on a substrate but that there also exists another factor that affects cell fate independently of growth or differentiation factors: cell shape^23–25^. The important role of cell shape on proliferation and differentiation has already been demonstrated for endothelial cells, muscle cells, hepatocytes, human mesenchymal stem cells and vascular smooth muscle cells^26–33^. Thus, controlling cell shape by implant surface properties may provide a promising approach to influence cell behaviour in a cell instructive manner, i.e. in a way that biomaterial-innate biophysical properties emerge as instructive factors for governing cell behavioural characteristics such as proliferation. This concept brings the cell-material interface and specifically the cell-material-crosstalk into focus. This crosstalk includes a variety of biomaterial features such as nano-/microtopography, surface wettability and mechanical properties, e.g. stiffness or force application, which co-control cell fate and functions. In this context it is noteworthy that despite their different nature, the aforementioned cues all affect cell behavioural adhesion events and, thus, affect the cytoskeleton crosstalk pathway^34^. Based on its fundamental role in controlling cell behaviour, incorporating cues for steering such cell–matrix crosstalk may be a promising strategy for engineering the next generation of implant surfaces with optimized tissue integration.

With respect to a structure function relationship between implant surface properties and osteoblast behaviour, the aim of the present study was to identify surface features of differently modified zirconia-based implant materials that influence cell attachment, morphogenesis and proliferation. Therefore, we first characterized the biomaterial surfaces in terms of surface topography, elemental composition and wettability. Osteoblast response was then analysed in terms of cellular morphology which was quantified by using a panel of previously recommended shape descriptors^35^ and proliferation which was investigated by the metabolic alamarBlue assay and by quantifying the DNA content at days 1 and 7. Moreover, by employing correlation analysis, we firstly examined potential correlations between implant-innate surface parameters and cell functions like morphogenesis and proliferation, and secondly between the cell functions themselves.

## Results

### Surface characterization

**Figure 1:**
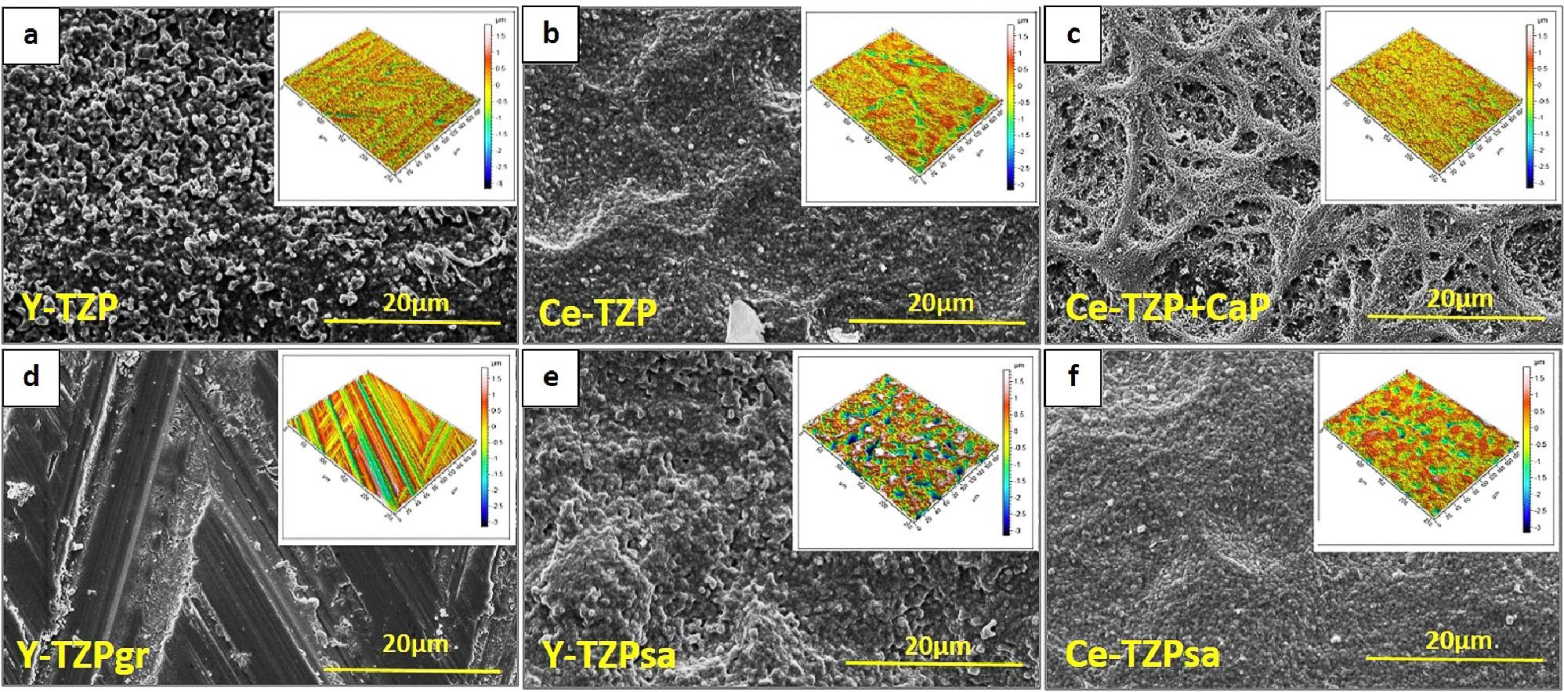
SEM micrographs and 3D reconstructed interferometer images (inserts) of (**a**) Y-TZP, (**b**) Ce-TZP, (**c**) Ce-TZP + CaP, (**d**) Y-TZPgr, (**e**) Y-TZPsa and (**f**) Ce-TZPsa. Magnification of SEM micrographs was set to × 2,000; measurement area of interferometric images was 200 µm × 260 µm. 3D reconstructed IFM images were created by the software MountainsMap Premium.

In order to characterize the differently modified zirconia surfaces with respect to their topographical and physicochemical properties, we employed scanning electron microscopy (SEM) and interferometry (IFM) to visualize and quantitatively grasp the surface topography, performed EDX analysis to describe the chemical composition and contact angle measurement to characterize the wettability of the biomaterial surfaces. SEM analysis revealed that the zirconia discs differed considerably in their surface structure, depending on the surface treatment (Fig. 1a–f). Y-TZP surfaces that were not further processed after sintering showed homogeneously distributed grainy peaks on the micrometre level (Fig. 1a, SEM), whereas on the macroscopic level the surfaces were crossed by shallow grooves (Fig. 1a, insert, IFM reconstructed image). By contrast, surfaces in this group additionally coated with Ce-TZP showed a dense and grainy structure with undulated depressions (Fig. 1b). Coating of Ce-TZP samples with CaP resulted in a thin layer with net-like structures, which was homogeneously distributed over the zirconia-based surfaces (Fig. 1c) and which was not present on Y-TZP + CaP surfaces. The formation of the net-like structures on Ce-TZP surfaces can be explained by the combination of the coating technique and the substrate used. A suspension with CaP powder was sprayed on the surfaces of the sintered Ce-TZP substrates. When the sprayed droplets dried on the substrate surface, the CaP particles rearranged and accumulated at the edge of the droplet where a thin circular wall of particles was formed. The net-like structure was then formed by randomly adding additional overlapping walls from other droplets. In contrast, the Y-TZP substrate was not sintered and the porous nature of the substrate removed the water from the droplets by absorption. Thus, there was no rearrangement of the CaP particles during drying on the Y-TZP surface, which contributed to an even distribution of the CaP particles. Y- and Ce-TZP discs, which had been grinded after sintering, exhibited smooth surface characteristics at the low micrometre level, and the surfaces were divided into little rhombi by straight parallel grooves and protrusions (Fig. 1d). Regarding the blasted materials, SEM micrographs revealed that the topography of the only-sintered Y-TZP surface was changed from a surface with grainy peaks to a surface with smoother and denser characteristics, which was devoid of aforementioned structures, by blasting (compare Fig. 1a,e). On the contrary, the micro-texture of the blasted Ce-TZP surface hardly changed when compared with untreated Ce-TZP (compare Fig. 1b,f). On the macroscopic level, however, both blasted materials displayed, unlike corresponding untreated surfaces, large dimples/pits in the surface (Fig. 1e,f, inserts).

**Figure 2:**
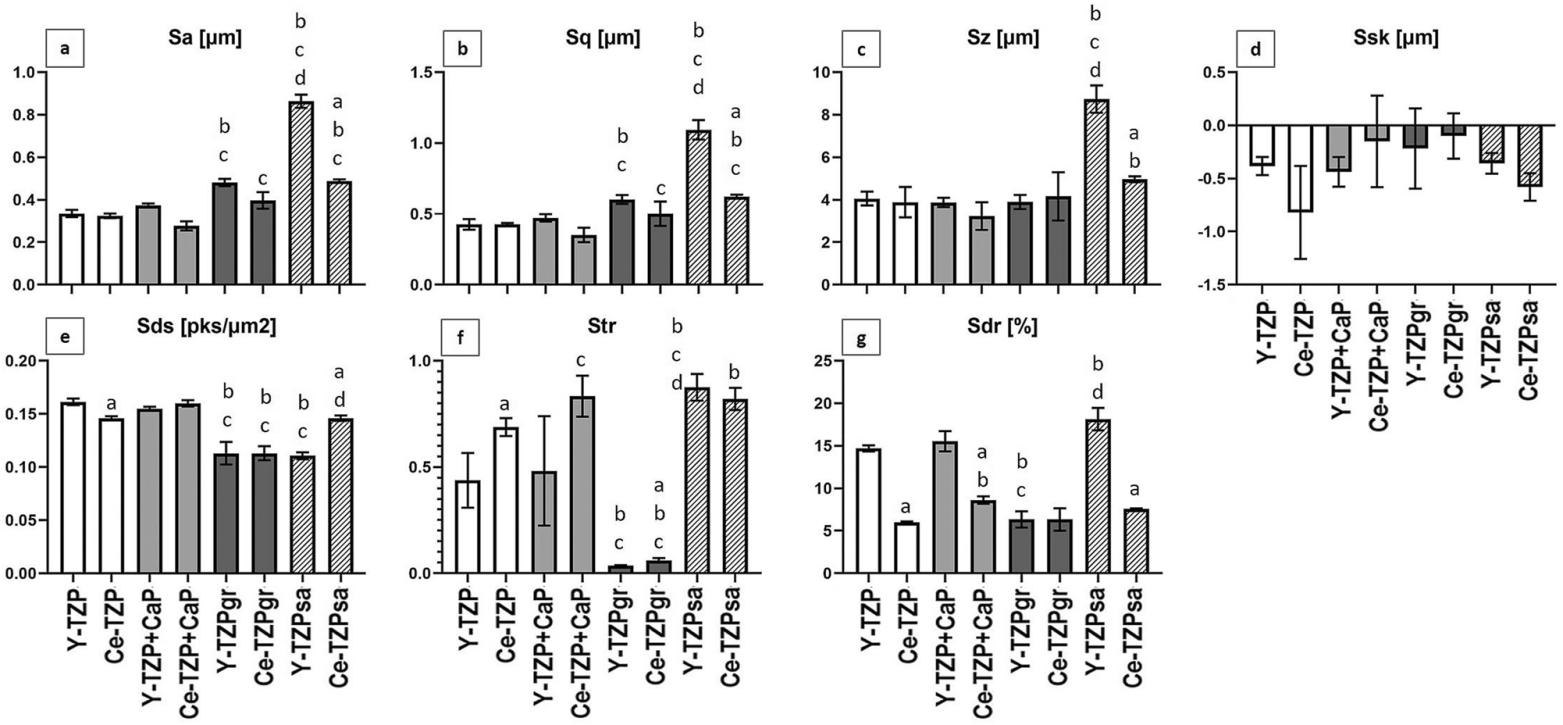
Quantitative surface characterization of zirconia biomaterials. Surface topography was evaluated by interferometry. Surface parameters describing the topography of implant surfaces were (**a**) S_a_ (average surface height deviation amplitude), (**b**) S_q_ (root-mean-square deviation), (**c**) S_z_ (ten-point height of surface topography), (**d**) S_sk_ (skewness), (**e**) S_ds_ (density of summits), (**f**) S_tr_ (texture aspect ratio) and (**g**) S_dr_ (surface enlargement compared to a totally flat reference area). Data show mean values of three different areas per specimen ± SEM (n = 3). Statistically significant differences (p < 0.05, Tukey’s HSD test) were marked with “a” if a Ce-TZP surface differed statistically significant from the corresponding Y-TZP surface, with “b” if Y-TZP + CaP, Y-TZPgr or Y-TZPsa differed from Y-TZP or if Ce-TZP + CaP, Ce-TZPgr or Ce-TZPsa differed from Ce-TZP, with “c” if Y-TZPgr or Y-TZPsa differed from Y-TZP + CaP or if Ce-TZPgr or Ce-TZPsa differed from Ce-TZP + CaP and with “d” if Y-TZPsa differed from Y-TZPgr or if Ce-TZPsa differed from Ce-TZPgr.

The quantitative analysis of surface topography by IFM (Fig. 2) demonstrated that the structural differences between the material groups were mainly reflected by the spatial and hybrid parameters S_tr_ (Fig. 2f) and S_dr_ (Fig. 2g). S_tr_ quantified the micro-anisotropy and strong directional structures of the grinded surfaces (S_tr_ = 0.04 and 0.06) as well as the uniform surface texture of Ce-TZP (S_tr_ = 0.69), Ce-TZP + CaP (S_tr_ = 0.83) and Y-/Ce-TZPsa (S_tr_ = 0.88 and 0.82). Surfaces with abundance of small peaks, like Y-TZP (Fig. 1a) and Y-TZP + CaP, or few but large height deviations as observed for Y-TZPsa (Fig. 1e, insert) yielded significant higher values for surface enlargement, as measured by S_dr_, than corresponding Ce-TZP samples with a denser surface texture aspect. In detail, S_dr_ values were 14.70%, 15.53% and 18.13% for Y-TZP, Y-TZP + CaP and Y-TZPsa, and 5.96%, 8.61% and 7.58% for Ce-TZP, Ce-TZP + CaP and Ce-TZPsa, respectively. The lower S_dr_ values of Ce-TZP-based surfaces suggest that the additional thin Ce-TZP layer filled the surface irregularities on the bulk Y-TZP discs after coating. By contrast, grinding resulted in Y- and Ce-TZP surfaces with similar topographical properties, which were characterized by a low surface enlargement of about 6% and a low peak density with S_ds_ = 0.11/µm^2^ (Fig. 2e). With regard to the latter parameter, blasting of Y-TZP exerted a similar smoothening effect, concerning the peak density, thereby yielding exactly the same S_ds_ values of 0.11/µm^2^ as determined for the grinded surfaces. Average surface roughness (S_a_ and S_q_) (Fig. 2a,b) and ten-point height of the surface (S_z_) (Fig. 2c) were less different between the material groups, with the exception of sandblasted Y-TZPsa. As already mentioned above, sandblasting of Y-TZP produced large height deviations, which were here reflected by the significantly higher amplitude parameters S_a_, S_q_ and S_z_ for Y-TZPsa, when compared to the other materials. The negative skewness of all surfaces as measured by S_sk_ (Fig. 2d) disclosed that all surfaces exhibited small peaks with comparatively deep and narrow valleys^36^. Based on the data obtained from SEM and IFM analysis it can be summarized that the applied surface treatments created distinct surface topographies, mainly related to the texture pattern (S_tr_) and surface enlargement (S_dr_). In detail, (i) coating of the bulk Y-TZP material with Ce-TZP and Ce-TZP + CaP caused a levelling of surface irregularities and hereby a reduction of the surface enlargement. (ii) Grinding yielded highly micro-anisotropic surfaces with smooth properties on the micrometre level in conjunction with a low degree of surface enlargement, whereas (iii) blasting increased the roughness, percentage of surface enlargement and isotropic properties of the treated surfaces.

Analysis of the chemical surface composition by EDX (Table 1) showed that the main components of all test surfaces were zirconium with 43–64 mass% and oxygen with 26–35 mass%. Furthermore, 4–6 mass% carbon were found on all surfaces. As was to be expected, the Y-TZP surfaces additionally consisted of 4–4.6% yttrium. Furthermore, sandblasted Y-TZPsa surfaces contained 2.46 mass% aluminium while on all other Y-TZP surfaces only traces of aluminium could be found. This indicates that the detected aluminium on the Y-TZPsa originated from the blasted aluminium oxide particles and thus represents residues from the surface modification process. The Ce-TZP surfaces consisted of a ceria-stabilized zirconia-alumina-strontium hexa-aluminate composite and, accordingly, exhibited in addition to aforementioned zirconium, oxygen and carbon, 6.6–8.7% ceria and 3.3–4.7% aluminium, whereas strontium was found inconstantly in traces on the samples (not specified in Table 1). Calcium (Ca) and phosphorus (P) were exclusively detected on calcium-phosphate coated surfaces at a Ca/P ratio of 1.8–1.9, or more specifically, 4.26 mass% Ca and 2.36 mass% P for Y-TZP + CaP and 7.35 mass% Ca and 3.83 mass% P for Ce-TZP + CaP. All other material groups displayed only traces of phosphorus on their surfaces. Regarding surface wettability, contact angle measurement revealed that with exception of Y-TZP and Y-TZPsa, all test surfaces displayed a contact angle below 90° and thus can be classified as hydrophilic biomaterials^37^ (Table 1). Within the hydrophilic biomaterial group the measured contact angles varied between 51.25° for the CaP-coated Ce-TZP to 85.93° for Ce-TZPsa. As surface wettability of implant materials is determined by the chemical composition and by structural surface features, the different hydrophilic properties of the test materials in the present work may originate from the various combinations of microtopography and surface chemistry.

**Table 1:** (Physico-)chemical characterization of zirconia biomaterials.

Biomaterial	Zr [mass%]	O [mass%]	Y [mass%]	Ce [mass%]	Al [mass%]	Ca [mass%]	P [mass%]	C [mass%]	Contact angle [°]
Y-TZP	64.0 ± 0.5	25.6 ± 0.1	4.52 ± 0.11	–	Traces	–	Traces	4.63 ± 0.66	97.86 ± 0.31
Ce-TZP	54.2 ± 0.6^a^	27.35 ± 0.3^a^	Traces	8.26 ± 0.12	4.36 ± 0.05	–	Traces	4.40 ± 0.82	68.35 ± 1.81
Y-TZP + CaP	55.7 ± 0.1^b^	28.01 ± 0.31^b^	4.07 ± 0.12	–	Traces	4.26 ± 0.13	2.36 ± 0.08^c^	4.95 ± 0.33	74.08 ± 2.61
Ce-TZP + CaP	43.2 ± 0.8^a,b^	30.5 ± 0.3^a,b^	Traces	6.63 ± 0.29^b^	3.26 ± 0.14^a,b^	7.35 ± 0.38^a^	3.83 ± 0.31^a,c^	4.67 ± 0.18	51.25 ± 0.83
Y-TZPgr	61.9 ± 0.4^c^	26.9 ± 0.1^b^	4.57 ± 0.24	–	traces	–	Traces	5.46 ± 0.17	75.66 ± 0.71
Ce-TZPgr	46.5 ± 0.6^a,b,c^	35.0 ± 0.0^a,b,c^	Traces	6.96 ± 0.19^b^	4.69 ± 0.04^a,c^	–	Traces	5.27 ± 1.01	80.77 ± 1.17
Y-TZPsa	60.5 ± 0.8^b,c^	28.5 ± 0.8^b,d^	3.94 ± 0.21	–	2.46 ± 0.14^b,c,d^	–	Traces	3.68 ± 0.76	95.64 ± 0.55
Ce-TZPsa	53.6 ± 0.4^a,c,d^	28.6 ± 0.3^b,c,d^	Traces	8.75 ± 0.06^c,d^	4.56 ± 0.10^a,c^	–	Traces	3.47 ± 0.38	85.93 ± 0.55

Elemental composition of the zirconia samples in mass% was measured by EDX and contact angles were calculated as a measure for surface hydrophilicity. Data show mean values ± SEM (n = 3 per group for EDX analysis and n = 10 per group for contact angle measurement). Statistically significant differences (p < 0.05, Tukey’s HSD test) were marked with “a” if a Ce-TZP surface differed statistically significant from the corresponding Y-TZP surface, with “b” if Y-TZP + CaP, Y-TZPgr or Y-TZPsa differed from Y-TZP or if Ce-TZP + CaP, Ce-TZPgr or Ce-TZPsa differed from Ce-TZP, with “c” if Y-TZPgr or Y-TZPsa differed from Y-TZP + CaP or if Ce-TZPgr or Ce-TZPsa differed from Ce-TZP + CaP and with “d” if Y-TZPsa differed from Y-TZPgr or if Ce-TZPsa differed from Ce-TZPgr.

### Cell morphology

Morphology of AO was analysed by SEM and fluorescence microscopy of red labelled actin cytoskeleton at days 1 and 7. In order to describe and quantify the cell morphology on the different biomaterial surfaces, we measured the cell area, perimeter and the major and minor axis of the cell body. In addition, we calculated the hybrid shape parameters roundness, circularity and aspect ratio.

**Figure 3:**
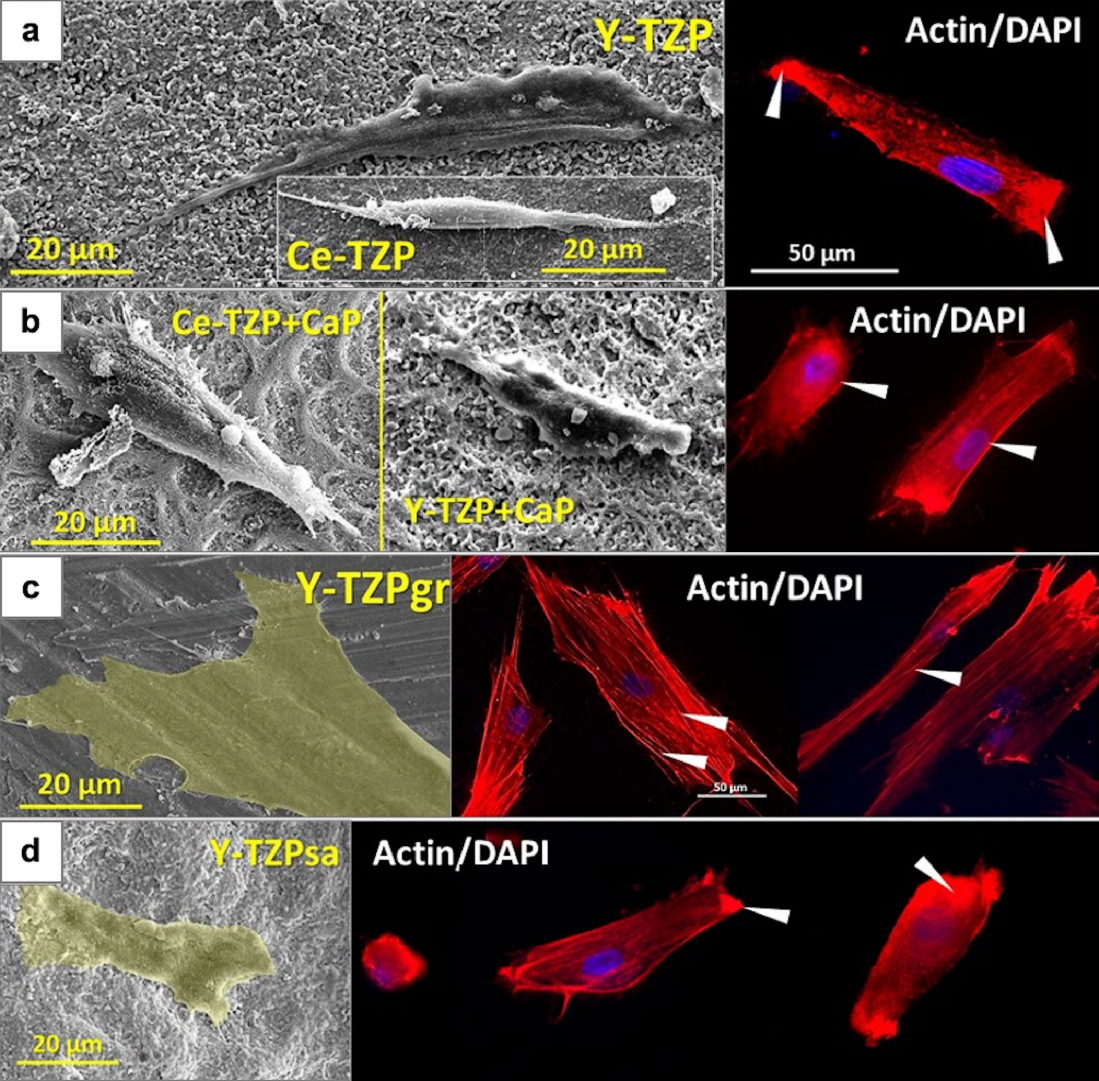
Representative SEM micrographs (left) and corresponding actin staining of AO (right) after one day of culture on (**a**) Y-/Ce-TZP, (**b**) Y-/Ce-TZP + CaP, (**c**) Y-TZPgr and (**d)** Y-TZPsa. SEM imaging parameters: EHT voltage level = 8.00 kV; magnification =  ×1,000. The actin cytoskeleton was labelled with red-fluorescent phalloidin and nuclear stain was performed with DAPI (blue fluorescence).

As shown in Fig. 3, AO grown on Y-/Ce-TZP for one day displayed an elongated spindle-shaped morphology and actin fluorescence mainly located at the apical cell borders (Fig. 3a, arrow heads), while actin stress fibres were absent. A similar situation was observed for AO on CaP-coated and sandblasted surfaces (Fig. 3b,d). By contrast, AO on grinded surfaces appeared considerably elongated and flattened, with clearly visible actin stress fibres mainly aligned parallel to the cells’ longitudinal axis within the entire cell body (Fig. 3c, SEM micrograph and fluorescence image, arrow heads).

**Figure 4:**
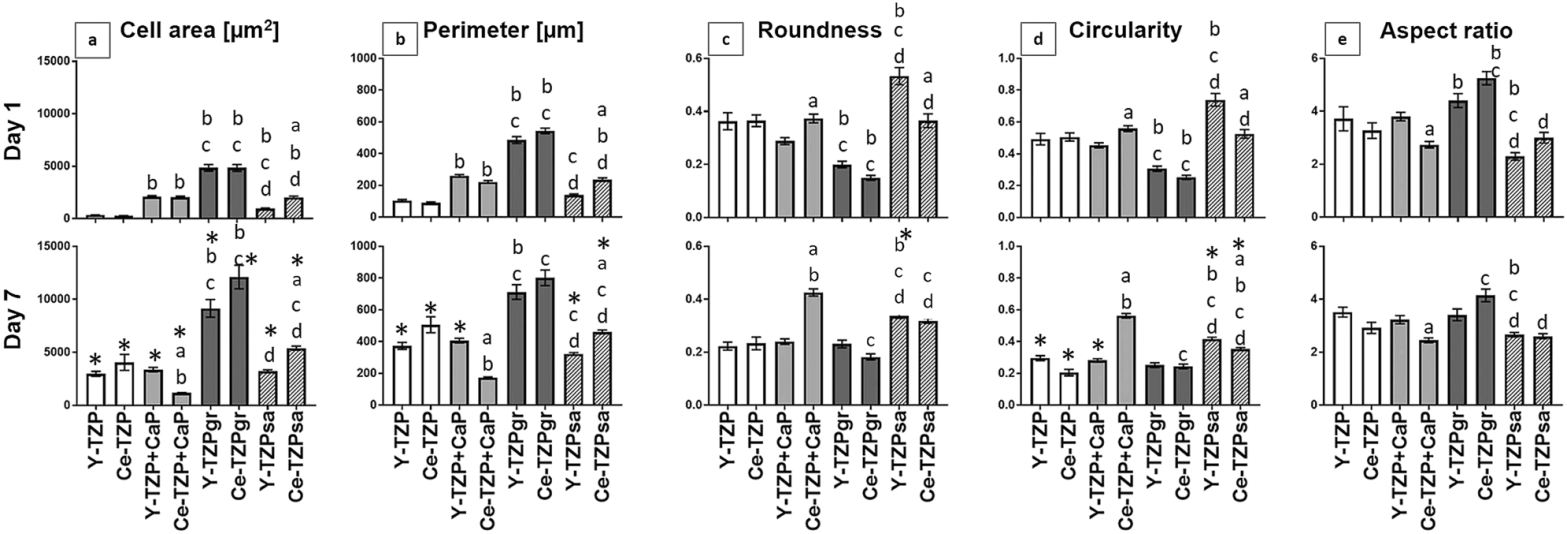
Quantitative morphometric analysis of osteoblast morphology after 1 and 7 days culture on zirconia-based implant surfaces with different surface topographies. Graphs show (**a**) cell area, (**b**) perimeter, (**c**) roundness, (**d**) circularity and (**e**) aspect ratio. Data is presented as mean values ± SEM (20 < n < 325). Statistically significant differences (p < 0.05, Dunn’s test) were marked with “a” if a Ce-TZP surface differed statistically significant from the corresponding Y-TZP surface, with “b” if Y-TZP + CaP, Y-TZPgr or Y-TZPsa differed from Y-TZP or if Ce-TZP + CaP, Ce-TZPgr or Ce-TZPsa differed from Ce-TZP, with “c” if Y-TZPgr or Y-TZPsa differed from Y-TZP + CaP or if Ce-TZPgr or Ce-TZPsa differed from Ce-TZP + CaP and with “d” if Y-TZPsa differed from Y-TZPgr or if Ce-TZPsa differed from Ce-TZPgr. *Statistically significant difference between day 1 and day 7.

The quantitative evaluation of the cell morphology, summarized in Fig. 4, confirmed these observations at day 1. AO on Y-/Ce-TZPgr revealed the highest values for cell area (4,852 and 4,843 µm^2^, Fig. 4a) and perimeter (486 and 544 µm, Fig. 4b) and high aspect ratio (4.41 and 5.25, Fig. 4e) coinciding with the lowest roundness values (0.2 and 0.15, Fig. 4c), which points to a highly elongated and flattened cell morphology. The low circularity values (0.31 and 0.25, Fig. 4d) on these surfaces indicate that the elongated morphology was accompanied by larger or more membrane protrusions compared with AO on the other biomaterials. By contrast, Y-/Ce-TZP and Y-TZPsa yielded lower values for cell area (Y-/Ce-TZP: 345 and 267 µm^2^, Y-TZPsa: 955 µm^2^), perimeter (Y-/Ce-TZP: 105 and 90 µm, Y-TZPsa: 139 µm) and aspect ratio (Y-/Ce-TZP: 3.72 and 3.27 µm^2^, Y-TZPsa: 2.3), while roundness (Y-/Ce-TZP: 0.36 and 0.37, Y-TZPsa: 0.53) and circularity (Y-/Ce-TZP: 0.49 and 0.51, Y-TZPsa: 0.74) were elevated compared with the other material groups. This suggests that AO displayed a least spread and more rounded morphology on Y-/Ce-TZP and Y-TZPsa. Regarding cell morphology at day 7, it is noticeable that on the Y-/Ce-TZPgr surfaces, roundness (0.23 and 0.18), circularity (0.25 and 0.24) and aspect ratio (3.41 and 4.15) remained fairly constant and cell area (9,150 and 12,112 µm^2^) even increased, thus suggesting that the elongated and spread cell shape persisted on these surfaces. In contrast, the other surfaces showed a stronger modulation of the aforementioned parameters at the analysed time points. In detail, on Y-/Ce-TZP and Y-/Ce-TZPsa cell area and perimeter increased, whereas circularity decreased from day 1 to day 7, roundness showed a trend towards lower values and the aspect ratio remained virtually unchanged. Concerning CaP-coated surfaces, spreading and shape parameters indicated that cell spreading on Y-TZP + CaP increased between day 1 and 7, whereas on Ce-TZP + CaP cell area and perimeter seemed to decrease. These findings, in conjunction with elevated values for roundness and circularity point to a rounding of the cells on Ce-TZP + CaP from day 1 to day 7.

Since quantitative morphometry demonstrated time- and biomaterial-dependent modulation of AO morphogenesis, we investigated possible correlations between cell shape, surface topography and wettability. For this purpose, we next performed a Spearman’s test for correlation between aforementioned cell morphology parameters and interferometric surface parameters as well as contact angles. The Spearman’s test for correlation provides values ranging from − 1 to 1, the so-called Spearman’s rho (r_s_), whereby values close to − 1 suggest an inverse correlation, values around 0 reject any correlation and values close to 1 indicate a strong correlation between two parameters. The results, summarized in Table 2, demonstrate that the topographic surface parameter S_tr_ correlated significantly with cell aspect ratio (p = 0.001 for day 1 and p = 0.011 for day 7), roundness (p = 0.007 for day 1 and p = 0.007 for day 7) and circularity (p = 0.001 for day 1, day 7 not significant), whereat the aspect ratio exhibited an inverse correlation. These correlations reveal that the more uniform the surface structure in all directions, i.e. isotropic was, the less elongated the cells were, and that lesser or smaller cellular protrusions were found. Cell perimeter showed inverse correlation with S_tr_ and S_dr_ at day 7 (p = 0.046 for both correlations), indicating that isotropic surface properties and high surface enlargement reduced cell perimeter.

**Table 2:** Values obtained for Spearman’s rho for the correlation of surface parameters and morphometric parameters.

Surface parameter	Cell area		Perimeter		Roundness		Circularity		Aspect ratio	
	d1	d7	d1	d7	d1	d7	d1	d7	d1	d7
S_a_	0.24	0.45	0.36	0.21	− 0.05	0.02	0.05	0.12	− 0.06	0.05
S_q_	0.23	0.49	0.35	0.25	− 0.01	0.05	0.06	0.07	− 0.06	0.01
S_z_	0.06	0.53	0.22	0.30	0.20	0.16	0.20	− 0.02	− 0.12	− 0.17
S_sk_	0.64	0.07	0.57	0.02	− 0.43	− 0.14	− 0.26	0.19	0.23	0.26
S_ds_	− 0.31	**− 0.69**	− 0.38	− 0.57	0.13	0.25	0.19	0.32	− 0.22	− 0.25
S_tr_	− 0.52	**− 0.60**	− 0.52	− 0.74*	0.88*	0.88*	0.95*	**0.69**	− 0.93*	− 0.86*
S_dr_	− 0.12	**− 0.62**	− 0.12	− 0.74*	0.26	0.43	0.43	**0.69**	− 0.49	− 0.19
Contact angle	− 0.21	0.05	− 0.07	− 0.02	− 0.05	− 0.31	0.02	0.17	− 0.02	0.36

Values pointing at high correlation are in bold; statistical significances (p < 0.05) are marked by asterisks.

Intriguingly, despite the aforementioned variations in hydrophilicity of the different biomaterials, no correlation between the surface wettability and cell morphology could be found. This indicates that the structural surface properties, or more precisely the surface topography, appeared to superimpose the impact of surface wettability on cell morphology.

### Cell proliferation

Since we detected biomaterial-associated differences in cell morphogenesis on the zirconia surfaces under study, we investigated how surface topography and/or chemistry influenced cell proliferation. Therefore, we next analysed the metabolic activity and proliferation of AO at days 1 and 7 on the zirconia discs. This was carried out by measuring the mitochondrial reduction of the redox indicator alamarBlue (AB) in the cell culture supernatant and the DNA content in the corresponding cell lysates.

**Figure 5:**
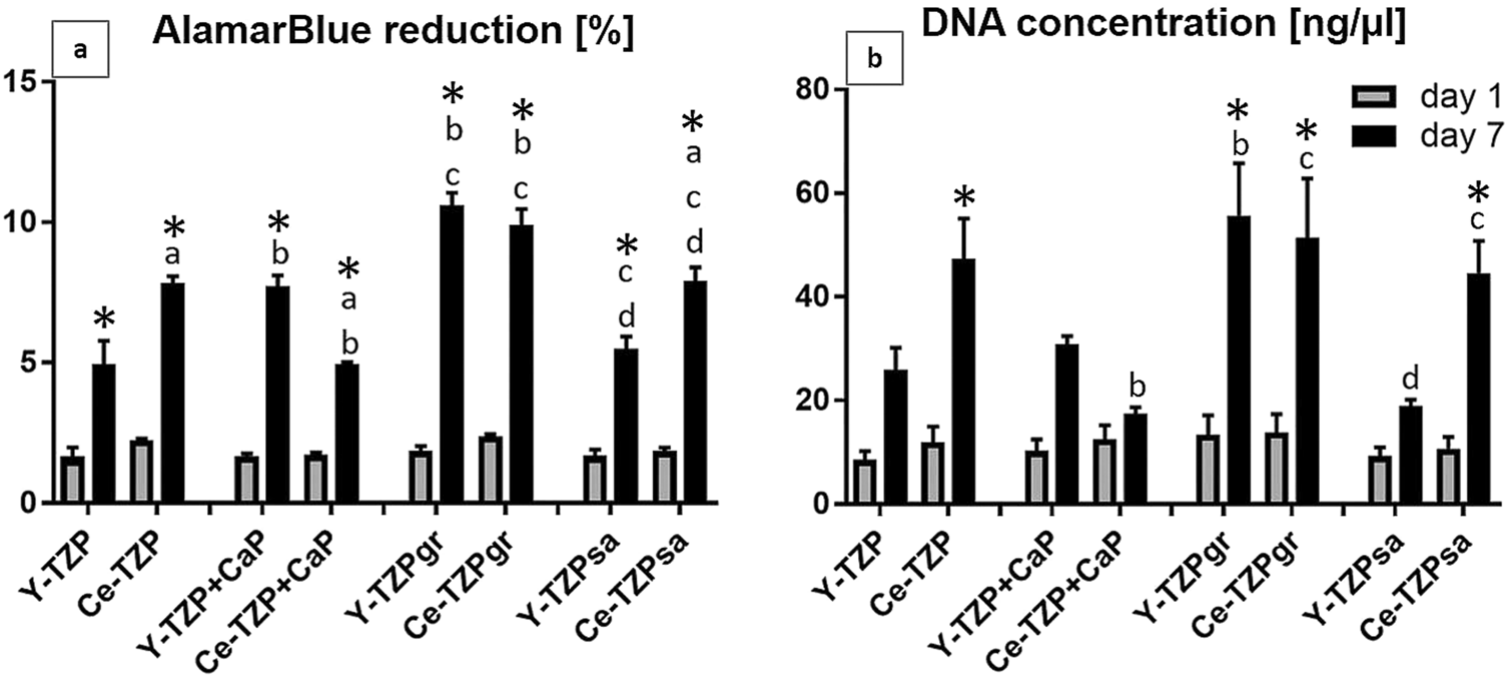
(**a**) AlamarBlue reduction and (**b**) DNA concentration of AO after 1 and 7 days of culture. Data show mean values ± SEM (n = 6 per group). Statistically significant differences (p < 0.05, Tukey’s HSD test) were marked with “a” if a Ce-TZP surface differed statistically significant from the corresponding Y-TZP surface, with “b” if Y-TZP + CaP, Y-TZPgr or Y-TZPsa differed from Y-TZP or if Ce-TZP + CaP, Ce-TZPgr or Ce-TZPsa differed from Ce-TZP, with “c” if Y-TZPgr or Y-TZPsa differed from Y-TZP + CaP or if Ce-TZPgr or Ce-TZPsa differed from Ce-TZP + CaP and with “d” if Y-TZPsa differed from Y-TZPgr or if Ce-TZPsa differed from Ce-TZPgr. *Statistically significant difference between day 1 and day 7.

As shown in Fig. 5, AB reduction (Fig. 5a) and DNA concentration (Fig. 5b) showed only slight differences between the samples at day 1, thus indicating that the initial attachment efficiency of the cells was comparable for all biomaterials. From day 1 to day 7 AB reduction increased significantly on all surfaces, albeit to differing degrees, with the highest values obtained on the grinded surfaces, namely Y- and Ce-TZPgr at day 7. Furthermore, it is striking that the Ce-TZP and Ce-TZPsa surfaces seemed to better support cell proliferation than their Y-TZP-based counterparts, as the AB reduction as well as DNA concentration showed a higher increase from day 1 to day 7 on these Ce-TZP surfaces with matched Y-TZP discs. More precisely, AB reduction on Ce-TZP/Ce-TZPsa at day 7 was 3.5/4.3 times higher than at day 1 and DNA concentration on these surfaces increased 4.0/4.2-fold during the experiment duration, while corresponding values for Y-TZP and Y-TZPsa were 3.0 and 3.2-fold for AB reduction, and 3.0 and 2.1-fold for DNA concentration. Such proliferation behaviour very likely arose from the different surface properties created by the Ce-TZP coating, mainly characterized by low surface enlargement (S_dr_) values (see also Fig. 2), rather than from the chemical composition or hydrophilic properties, since on grinded surfaces, which displayed the smoothest properties on cellular level, cell growth showed a similar trend on Y- and Ce-TZP.

The test for correlation between proliferation, surface topography and wettability substantiated this assumption, as the surface enlargement (S_dr_) correlated inversely with the AB reduction at day 1 (p = 0.011) and with the DNA concentration at day 7 (p = 0.037), with the same trend at day 7 (p = 0.096) and 1 (p = 0.058), respectively (Table 3). Furthermore, the surface parameter S_tr_ was also associated with cell proliferation at day 7, since Spearman’s rho yielded values of − 0.62 (p = 0.115) for the AB reduction, and − 0.76 (p = 0.037) for the DNA concentration. This means that the more isotropic a surface was the lower was AO proliferation. Hence, the results of the statistical analysis demonstrate that surface properties including anisotropy and low surface enlargement better supported cellular proliferation on zirconia discs. In contrast, there was no correlation between the examined cell proliferation parameters and measured contact angles.

**Table 3:** Values obtained for Spearman’s rho for the correlation of surface parameters and proliferation parameters.

Surface parameter	AB reduction		DNA concentration	
	d1	d7	d1	d7
S_a_	0.02	0.48	− 0.12	0.24
S_q_	0.08	0.51	− 0.07	0.28
S_z_	0.40	0.49	0.14	0.26
S_sk_	0.21	0.02	0.52	− 0.02
S_ds_	**− 0.63**	**− 0.69**	− 0.44	− 0.57
S_tr_	− 0.31	**− 0.62**	− 0.40	− 0.76*
S_dr_	− 0.86*	**− 0.64**	**− 0.71**	− 0.76*
Contact angle	− 0.24	0.02	− 0.55	− 0.05

Values pointing at high correlation are in bold; statistical significances (p < 0.05) are marked by asterisks.

### Correlations between cell morphology and proliferation

**Figure 6:**
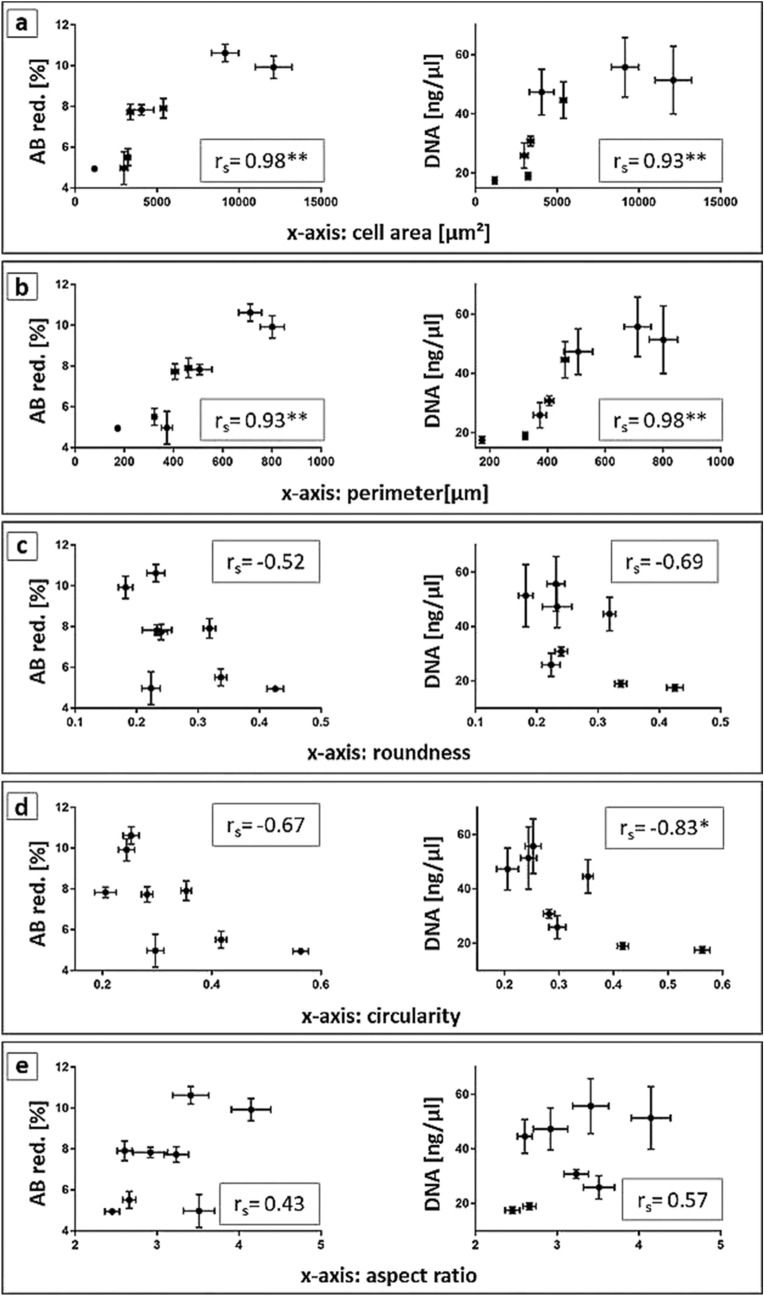
Values obtained in proliferation experiments at day 7 (y-axis) plotted versus data of morphometric analysis (x-axis), more precisely (**a**) cell area, (**b**) perimeter, (**c**) roundness, (**d**) circularity and (**e**) aspect ratio. Values for Spearman’s rho are listed at the lower right corner of each graph. Values for Spearman’s rho were marked with one asterisk if the p-value was lower than 0.05 and with two asterisks for p-values smaller than 0.01.

The data obtained from cell morphogenesis and proliferation experiments on the different zirconia surfaces revealed that AO with an elongated and spread morphology at day 1 showed a higher increase in metabolic activity and DNA concentration, and thus cell number, up to day 7, compared to less spread cells with higher roundness and circularity values (compare grey and striped columns of Ce-TZP + CaP and Y-TZPsa with dark grey columns in Fig. 4 for morphology and the columns for these surfaces in Fig. 5 for DNA concentration). Therefore, values for AB-reduction and DNA concentration on Ce-TZP + CaP and Y-TZPsa surfaces were lower compared with e.g. grinded biomaterials at day 7. This observation suggests a direct link between cell morphology and proliferation. To visualize such correlation, we plotted the values detected for AB-reduction and DNA concentration versus the examined cell morphology parameters, i.e. cell area, perimeter, roundness, circularity and aspect ratio, and calculated the Spearman’s rho for each parameter. Figure 6 shows the scatter diagrams with the calculated Spearman’s rho (r_s_). Scatter diagrams and Spearman’s correlation test revealed a strong and statistically significant correlation of AB-reduction as well as of DNA concentration with cell spreading as measured by cell area (Fig. 6a) and perimeter (Fig. 6b) at day 7 (r_s_ = 0.98, p = 0.0004 for the correlation of AB-reduction and cell area, r_s_ = 0.93, p = 0.002 for the correlation of AB-reduction and perimeter, r_s_ = 0.93, p = 0.002 for the correlation of DNA concentration and cell area and r_s_ = 0.98, p = 0.0004 for the correlation of DNA concentration and perimeter). Corresponding values for circularity (Fig. 6d) were: r_s_ = − 0.67 (p = 0.083) and r_s_ = − 0.83 (p = 0.015). Hence, osteoblast spreading apparently favoured the metabolic activity and proliferation while circularity was associated with lower DNA concentration. Roundness (Fig. 6c) and aspect ratio (Fig. 6e) did not show statistically significant correlation with AB-reduction nor DNA concentration. Spearman’s rho obtained for these correlations seemed to confirm that cell elongation favoured proliferation while cellular roundness decreased proliferation. Scatter diagrams furthermore revealed a switch in proliferation behaviour that occurred by surpassing a critical cell area of approximately 3,000–4,000 µm^2^ or 400–500 µm cell perimeter, respectively.

## Discussion

In our previous studies we demonstrated that implant surface topography and chemistry influence bone cell response in vitro and in vivo^6,38,39^, thereby pointing to cell instructive biomaterial properties, which emerge from implant-inherent features. Based on these findings, we here investigated whether specific topographical and/or chemical surface features of differently modified zirconia-based implant materials can be correlated to alterations in cell morphogenesis and proliferation. In this context, our experimental set-up utilized a causal approach, in which implant surface properties were used to induce various cell morphologies. Correlation analyses were subsequently applied to determine the geometrical aspects of morphology that were affected by the chosen surface properties. In addition they were employed to determine the relationship between osteoblast morphology and their proliferation behaviour. The decisive role of the induction of a certain cell morphology on other cell behavioural features like directing the differentiation of mesenchymal stem cells, has been shown by Kilian and co-workers^30^. In a comparable approach, Rolauffs et al. have also demonstrated that specific surface properties can be utilized causally for inducing specific aspects of cell behavioural functions^35^. With respect to surface topography, the characterization of the roughened zirconia surfaces by SEM and interferometry revealed that the applied surface modification methods created distinct differences in the microtopographies, which were mainly reflected by the spatial and hybrid parameters surface texture aspect ratio S_tr_ and surface enlargement S_dr_. By combining IFM and SEM analysis for 3D-microtopography interpretation, we demonstrated that the micrometre-scale roughness parameter S_a_, an accepted predictive parameter for a successful osseointegration^5^ and thus the most commonly applied topographical parameter to characterize dental implant surfaces was not suitable to describe the surface microtopography with which the cells interact, but rather seemed to reflect surface characteristics at a supracellular level. This was exemplified by the grinded Y-TZPgr materials, which exhibited a higher S_a_-value than the untreated Y-TZP, but, from the cell’s viewpoint, displayed smoother surface characteristics (less rougher surfaces) at the low micrometre level than Y-TZP (compare SEM images Fig. 1a,d). Therefore, S_a_ and/or R_a_ may be unsuitable to describe an implant surface on a cell-instructive level, rendering them unreliable predictors for the outcome of cell morphogenesis and proliferation.

To find out which of the examined surface structures influenced cell behaviour in terms of morphogenesis and proliferation, we next demonstrated that the surface texture aspect ratio S_tr_, a measurement for surface isotropy, affected almost all cell shape parameters, except cell area, under study, and thus overall AO morphogenesis. In contrast, the hybrid parameter S_dr_ exerted its impact exclusively on cell spreading and the formation of cellular protrusions. However, osteoblast morphology remained unaffected by average surface roughness (S_a_). Similar findings were reported by Anselme et al. who, in the context of an implant surface, stated that “human osteoblasts are more sensitive to the organization and morphology of the roughness rather than to its amplitude”^14^. Our data thereby showed that isotropic surface structures and high surface enlargement induced less elongated and spread cell morphologies with less and/or smaller cellular protrusions. On the contrary, AO on anisotropic surfaces aligned preferentially along the axis of the surface grooves, and displayed elongated and spread morphologies with pronounced actin stress fibre formation. The latter cell response on the grinded surfaces may be caused by contact guidance, i.e. the alignment of cells along anisotropic surface features, and the smooth microtopography, which provided large bio-adhesive areas for the cells. In both cases, namely anisotropic and smooth surface properties, human MSCs and osteoblasts have been shown to form stable and large focal adhesions with large actin bundles, substantiated by stress fibres^40–42^, as also observed in the present study.

With respect to AO proliferation, again both aforementioned surface parameters S_tr_ and S_dr_ were shown to be linked with the metabolic activity and cell number. Here it became apparent that anisotropic surfaces with low surface enlargement generally favoured cell proliferation, whereas the proliferation rate decreased with increasing surface isotropy. Thus, our results demonstrate that both surface parameters S_tr_ and S_dr_ appear to be suitable to predict AO behaviour with respect to morphology and proliferation on implant surfaces in vitro and could be considered as cell instructive surface features during implant processing. The lack of correlation between S_a_/S_q_ and cell behavioural parameters found in this investigation together with our recently published report, which describes different cell and tissue reactions to implant surfaces with similar roughness values but different surface micro-architecture^6^, thereby add to the body of evidence that a surface specification by S_a_ and/or R_a_ may not allow reliable conclusions on osteoblast behaviour.

As the applied ceramic implant surfaces exhibited different chemical properties, we further characterized the elemental composition and wettability of the biomaterial surfaces to assess the potential impact of these material parameters on osteoblast response. However, our analysis revealed no significant effect of surface chemistry and wettability on cell functions, since surfaces with different elemental composition but comparable topography, such as Y-TZPgr and Ce-TZPgr, showed similar osteoblast behaviour, and no correlation between surface wettability and examined cell function parameters could be proven. This suggests that the effect of surface topography on osteoblast function seemed to overcompensate the impact of elemental surface composition and surface wettability. This assumption is supported by our earlier work which demonstrates that osteoblast functions are rather controlled by surface architecture than by (physico-)chemical implant surface properties in vitro^38^. With respect to the CaP-coated Ce-TZP surfaces that yielded the least spread AO morphology over culture time and low proliferation, it appeared that again the distinct micro-topography with its net-like structures observed by SEM may have caused the aforementioned osteoblast response. This would also be in line with other studies, reporting that the biological activity of CaP coatings mainly depends on the topography and crystallinity of the coating and can therefore lead to conflicting results, namely positive as well as negative biologic effects, depending on the properties of aforementioned variables^43^.

Our correlation analysis not only revealed surface topography inherent characteristics of dental implant surfaces as being cell-instructive but also substantiated the assumption that osteoblast proliferation depends on cell morphology. Focusing on the latter issue, a rounded and less spread cell shape as found on the CaP coating coincided with a low proliferation, whereas a spread and elongated morphology on anisotropic grinded surfaces was linked to an enhanced proliferation. The afore described correlation of cell spreading and proliferation has already been reported for cells of other tissue origin, namely hepatocytes, endothelial and smooth muscle cells, showing that cell spreading is essential for the survival and proliferation of adhesion-dependent cells^26–33^. With respect to this issue, our data analysis thereby not only provides evidence for a direct correlation between AO morphogenesis and proliferation, but further revealed a switch in proliferation behaviour by surpassing a critical cell size, or more specifically cell area, of approximately 3,000 to 4,000 µm^2^. Intriguingly, such an increase in cell proliferation has also been observed for bovine and human endothelial cells^26,27^ and human smooth muscle cells^32^, after exceeding a cell spreading area of approximately 1,500–2,200 µm^2^ and 500 µm^2^, respectively. This correlation together with aforementioned previous reports corroborates the existence of a cell-innate cell area threshold, which may function as a proliferative stimulus^32^. From a more abstract viewpoint, this could imply that cell morphology-instructive biomaterial features can be systematically used as proliferation triggers. Regarding the cytoskeletal involvement in the regulation of cell adhesion, it has been demonstrated for endothelial cells that herein, the actin cytoskeleton is involved in the morphogenesis-related cell spreading, and that actin stress fibre formation, similar to that seen for our AO on grinded surfaces, increases the cells’ mitotic activity^44,45^.

Taken together, for the here applied ceramic dental implant surfaces, that were subjected to commonly applied surface modification methods, topographical but not chemical/physicochemical surface parameters appeared to be cell instructive. This initial characterization of cell instructive parameters appears motivating, to further characterize implant-innate features which act cell instructive by controlling cell fate decisions. Thus, with respect to a putative translation of cell instructive parameters into the development of next generation biomaterials, cell instructive implant surfaces should for instance consider the aforementioned surface parameters (i) isotropy and (ii) surface enlargement. This is because if a biomaterial of interest should explicitly favour cell spreading and proliferation, the biomaterials surface features should include anisotropic properties in conjunction with low surface enlargement. These two parameters per se favour AO spreading, thereby considering that the aspect of spreading is a prerequisite for proliferation. In this context, the underlying molecular mechanisms of the material-cytoskeleton-crosstalk controlling cell fate and functions need further elucidation^46^.

At authoring this manuscript, techniques to imprint nanometre-scale features onto implant surfaces remained far from being integrated in commercially available implants and no established classification concepts of implant surfaces at the nanometre-level existed. The authors of this manuscript therefore recommend that future research focuses on the identification of topographical and/or physicochemical cell-instructive implant surface parameters at the nano- and micrometre-level and on their in vivo validation. This might render the design of implant surfaces with predictable influence on cell behaviour and subsequently osseointegration possible.

## Conclusions

The current study demonstrates that among the tested implant surface parameters, characterized for various zirconia-based implant biomaterials, texture aspect, and surface enlargement are significantly more effective than surface roughness or wettability in controlling morphogenesis and proliferation of AO in vitro. Regarding morphogenesis, calculation of cell area firstly reveals proliferation as a function of morphogenesis, and secondly, corroborates together with other reports the existence of a cell area-dependent threshold, which may function as cell-innate proliferation trigger. Moreover, the coincidence of a proliferation-favourable AO morphogenesis with actin stress fibres supports the notion that their presence can be used as cytoskeletal proliferation indicator. Based on the detected proliferation-supportive cellular responses towards certain implant surface features it can be concluded that they may in turn be used as cell behavioural predictors, which may be used as cell instructive parameters to be implemented in the next generation of “cell instructive” biomaterials.

## Materials and methods

### Implant materials and surface treatment

Biomaterial discs (15 mm in diameter, 1.5 mm thickness) used for cell culture were prepared from tetragonal zirconia polycrystals containing 3 mol% of yttrium oxide (Y-TZP) by uniaxial pressing. In order to compare the effects of the chemical and topographical surface properties on cell behaviour, different surface modification techniques were applied to the Y-TZP-discs. Biomaterials were grouped as follows: (i) sintered Y-TZP not further processed (referred to as Y-TZP), (ii) Y-TZP discs coated with a novel ceria stabilized zirconia-alumina-strontium aluminate nano-composite powder (referred to as Ce-TZP; see^47^ for detailed description), (iii) Y-TZP and Ce-TZP discs coated with a thin layer of calcium phosphate (referred to as Y-TZP + CaP and Ce-TZP + CaP, respectively; CaP = calcium phosphate), (iv) grinded Y-TZP discs (referred to as Y-TZPgr; gr = grinded), (v) Y-TZPgr discs coated with Ce-TZP (referred to as Ce-TZPgr), (vi) alumina sandblasted Y-TZP discs (referred to as Y-TZPsa; sa = sandblasted), and (vi) Y-TZPsa discs coated with Ce-TZP (referred to as (Ce-TZPsa). The discs for cell culture experiments were sterilized using low-temperature hydrogen peroxide gas plasma sterilization before performing the cell culture experiments.

### Surface characterization

Surface topography of the implant materials was examined as already described in a previous report^6^ by scanning electron microscopy (SEM, LEO435VP scanning electron microscope, Zeiss, Oberkochen, Germany) with the backscattered electron imaging mode and an accelerated voltage of 8.00–12.00 kV after sputter coating with gold–palladium for 60 s at 60 mA (SCD050; Balzers, Liechtenstein), and by light interferometry (IFM, MicroXAM 100 h; ADE; Phase Shift Technology, Tucson, USA). 3D reconstructed IFM images were created by the software MountainsMap Premium, version 7.4 (Digital Surf SARL, Besanҫon, France). The measuring area for IFM was set to 260 × 200 µm (n = 3 per group). Before parameter calculation, a digital (Gaussian) filter of 50 × 50 μm was applied to remove errors of form and waviness. Amplitude, spatial and hybrid parameters were obtained via IFM measurements to characterize the biomaterial surfaces in three dimensions (for detailed information about surface parameters see^48,49^. Surface amplitude was characterized by the parameters S_a_, S_q_, S_z_ and S_sk_. The main roughness parameter S_a_ describes the arithmetic mean value of the absolute surface asperity departures in μm and the root-mean-square deviation S_q_ the standard deviation of S_a_. Although S_a_ and S_q_ describe similar surface features both parameters are listed in this work since S_a_ is the most commonly used parameter to characterize surface roughness in medical science while S_q_ is preferred by statisticians^48,49^. Furthermore, surface amplitude was measured by the ten-point height of surface topography S_z_ which represents the mean value of the five highest peaks and five deepest valleys of a surface in micrometre and S_sk_ as a measurement for the asymmetry of surface deviations from the mean plane. S_sk_ adopts negative values if surface height distribution is shifted towards a predominance of valleys while positive values indicate that the surface height distribution has more peaks than valleys. Spatial parameters were the summit density S_ds_ describing the number of peaks per area in 1/mm^2^ and the texture aspect ratio of a surface designated as S_tr_. The latter parameter describes the topographic texture pattern and takes values between 0 and 1, with small values indicating strong anisotropy, i.e. less uniformity, and large values indicating uniform texture aspect in all directions^49^. The hybrid parameter S_dr_ (developed interfacial area ratio) was examined to describe the surface enlargement compared to a totally flat reference area and is given in percent. Elemental composition of the biomaterial surfaces was analysed by energy-dispersive X-ray spectroscopy (EDX) (n = 3 per group). EDX was performed with a JSM-IT100 (JEOL Ltd, Tokyo, Japan). Magnification was set to 1,000, excitation energy to 15 kV and time of measurement to 100 s. Surface wettability of the zirconia discs was evaluated by static contact angle measurement (n = 10 per group). Contact angles of 2 µl water droplets were analysed using the Dataphysics OCA 10 optical contact angle measuring system (Dataphysics GmbH, Filderstadt, Germany) by measuring the angles of each drop against the surface.

### Isolation and cultivation of primary osteoblasts

Alveolar bone osteoblasts (AO) were prepared from bone explants obtained during implant site preparation procedure at the Department of Prosthetic Dentistry, University of Freiburg, Germany. The collection and usage of the primary osteoblasts for scientific purposes was approved by the Ethics Committee of the Albert-Ludwigs-University, Freiburg, Germany (vote Nr. 411/08_121010) and informed consent was given by the patient. Research was performed in accordance with relevant guidelines and regulations. The bone specimens were dissected aseptically and cleaned in phosphate-buffered saline (PBS) to remove residues of blood and soft and/or bone marrow tissue. In order to ensure sterility of the bone explant cultures, the cleaned bone specimens were additionally sterilized in an iodide solution, subsequently washed with PBS. The bone explants and isolated cells were cultured in Dulbecco's Modified Eagle's Medium supplemented with 2% (w/v) glutamine, 10% (w/v) fetal calf serum and 50 µg/ml kanamycin, and maintained in a humidified 37 °C incubator with 5% CO_2_. All experiments were carried out with osteoblasts of passages 6 and 7.

### Evaluation of osteoblast morphology

Cell morphology of AO on different zirconia surfaces under study was analysed by SEM and fluorescence-based actin cytoskeleton staining with Texas Red-X-labelled phalloidin (Life Technologies, Darmstadt, Germany) as described earlier in Altmann et al.^6^. In detail, cells were fixed with 4% formaldehyde (PFA) in PBS for 20 min at room temperature after 1 and 7 days of culture. In addition to actin cytoskeleton staining, osteoblasts were analysed by SEM at day 1. Therefore, cells were fixed and dehydrated in an ascending ethanol series (ranging from 30 to 100% ethanol, three times each for 20 min at room temperature), critical point dried (CPD030 Critical Point Dryer, Bal-Tec AG, Balzers) and sputter coated with gold–palladium for 60 s at 60 mA (SCD050, Balzers). For actin staining, the samples were fixed at day 1 and 7, treated with 2% (w/v) bovine serum albumin in PBS and 0.2% TritonX-100 in PBS for 15 min, and 2% (w/v) BSA in PBS for further 15 min. Actin labelling was then performed by incubating the samples for 30 min with phalloidin conjugate diluted 1:40 in PBS containing 0.5% (w/v) BSA. Nuclei were stained with 300 nM DAPI for 15 min. Optical evaluation was performed with the fluorescence microscope Biozero BZ-8000 (KEYENCE, Neu-Isenburg, Germany) and quantitative morphometric analysis (20 < n < 325) was carried out with the microscope software BZ Analyzer II from KEYENCE. AO morphology was estimated by measuring the cell area and perimeter which both provide information on the extent of cell spreading^50^, the major axis representing the long axis of the smallest rectangle drawn around the cell body and the minor axis, presenting the rectangle width. In order to measure changes in cell shape we used in analogy to Uynuk-Ool and coworkers^35^ a panel of specific shape descriptors derived from the morphometrically collected data to calculate the hybrid morphology parameters roundness (4 × area / (π  ×  major axis)^2^), circularity (4 π × area / perimeter^2^) and aspect ratio (major axis / minor axis). High values for the roundness parameter indicate a high degree of cell roundness, whereas a high aspect ratio points to an elongated cell shape. The parameter circularity describes the change from a circle with a large number of protrusions into a circle without protrusions, i.e. specifies whether cellular protrusions are formed or not. A high value indicates the absence of cellular protrusions.

### Evaluation of metabolic activity and cell proliferation

The metabolic activity of AO cultured on the test surfaces was analysed by the alamarBlue (AB) metabolic assay (MorphoSys AbD, Düsseldorf, Germany). After 1 and 7 days of culture on the test surfaces the cells were incubated for 2 h at 37 °C with culture medium, supplemented with 10% (w/v) AB reagent (n = 6 per test group). Subsequently, the supernatant was analysed by fluorimetry according to the manufacturer’s instructions (Tecan, Männedorf, Germany). The percentage of AB reduction in the samples was calculated using a 100% reduced AB control as reference.

Proliferation of AO cultivated on the different zirconia discs was evaluated after 1 and 7 days of culture by performing DNA quantification of the samples with the Quant-iT PicoGreen (Invitrogen, Carlsbad, U.S.A.) assay according to the manufacturer’s protocol (n = 6 per group). Prior to DNA quantification cells were washed once with PBS and lysed by a freeze–thaw cycle at − 80 °C in 400 μl TE-buffer (10 mM Tris–HCl, 1 mM EDTA, pH 7.5)^38^.

### Statistical analysis

Differences between test groups were examined for statistical significance using the one-way ANOVA followed by a Tukey’s HSD post hoc test for normally distributed data (according to the Shapiro–Wilk-test for normality) and the Kruskal–Wallis ANOVA followed by a Dunn’s post hoc test for non-normally distributed data. To test for a possible correlation between surface parameters, cell morphometry and proliferation, the Spearman’s rank correlation test was applied. The Spearman’s test for correlation provides values ranging from − 1 to 1, the so-called Spearman’s rho (r_s_), whereby values close to − 1 suggest an inverse correlation, values around 0 reject any correlation and values close to 1 point at strong correlation between two parameters. The deviation of the Spearman’s rho from zero was examined for statistical significance using a two-tailed t-test.

## Data Availability

The datasets generated during and/or analysed during the current study are available from the corresponding author on reasonable request.
